# Vitamin D levels and their associations with survival and major disease outcomes in a large cohort of patients with chronic graft-vs-host disease

**DOI:** 10.3325/cmj.2016.57.276

**Published:** 2016-06

**Authors:** Mašenjka Katić, Filip Pirsl, Seth M. Steinberg, Marnie Dobbin, Lauren M. Curtis, Dražen Pulanić, Lana Desnica, Irina Titarenko, Steven Z. Pavletic

**Affiliations:** 1 Experimental Transplantation and Immunology Branch, NCI, NIH, Bethesda, MD, USA; 2 Jamnica d.d., Zagreb, Croatia; 3 Biostatistics and Data Management Section, NCI, NIH, Rockville, MD, USA; 4 Clinical Nutrition Department, Clinical Center, NIH, Bethesda, MD, USA; 5 Division of Hematology, Department of Internal Medicine, University Hospital Center Zagreb, Zagreb, Croatia; 6 Medical School University of Zagreb, Zagreb, Croatia; 7 Faculty of Medicine Osijek, J.J. Strossmayer University of Osijek, Osijek, Croatia; 8 Center for Cancer Research, NIH, Bethesda, MD, USA

## Abstract

**Aim:**

To identify the factors associated with vitamin D status in patients with chronic graft-vs-host disease (cGVHD) and evaluate the association between serum vitamin D (25(OH)D) levels and cGVHD characteristics and clinical outcomes defined by the National Institutes of Health (NIH) criteria.

**Methods:**

310 cGVHD patients enrolled in the NIH cGVHD natural history study (clinicaltrials.gov: NCT00092235) were analyzed. Univariate analysis and multiple logistic regression were used to determine the associations between various parameters and 25(OH)D levels, dichotomized into categorical variables: ≤20 and >20 ng/mL, and as a continuous parameter. Multiple logistic regression was used to develop a predictive model for low vitamin D. Survival analysis and association between cGVHD outcomes and 25(OH)D as a continuous as well as categorical variable: ≤20 and >20 ng/mL; <50 and ≥50 ng/mL, and among three ordered categories: ≤20, 20-50, and ≥50 ng/mL, was performed.

**Results** 69 patients (22.3%) had serum 25(OH)D ≤20 ng/mL. Univariate analysis showed that supplement intake, nutritional status (severely malnourished, moderately malnourished, well-nourished), race (African-American, other), and estimated creatinine clearance (eCCr) were associated with 25(OH)D levels. A predictive model was developed based on supplement intake, nutritional status, race, and eCCr, accurately predicting 77.9% of patients with 25(OH)D ≤20 and 65.2% of those with 25(OH)D >20 ng/mL. No association was found between vitamin D and major cGVHD characteristics, but patients with 25(OH)D ≤20 ng/mL had somewhat decreased survival.

**Conclusion** Nutritional status and adequate supplementation are important to maintain 25(OH)D >20 ng/mL in cGVHD patients. Intervention studies and more research is needed to reveal the underlying mechanism of vitamin D metabolism in cGVHD setting.

Humans get vitamin D in two major forms: cholecalciferol, which can be obtained when the skin is exposed to solar UV-B radiation and from a few animal-based foods, and ergocalciferol, which is obtained from dietary sources (1). Both forms are found in vitamin D supplements and fortified foods. Vitamin D from the skin and diet is metabolized in the liver to 25-hydroxivitamin D (calcidiol) [25(OH)D], which is considered to be the most useful marker of vitamin D status, which incorporates endogenous synthesis from solar exposure, dietary intake from foods, fortified products, and supplements (2). 25(OH)D is further converted via the action of 1-α-hydroxylase primarily in the kidney to 1,25-dihydroxyvitamin D (calcitriol) [1,25(OH)2D], the physiologically active form of vitamin D (1).

Targeting various immune cells, including monocytes, macrophages, dendritic cells, and T- and B-lymphocytes, vitamin D has a significant role in the maintenance of immune homeostasis (3), and its deficiency has been associated with a higher susceptibility to autoimmune diseases (4). Since chronic graft-vs-host disease (cGVHD) is a multi-organ alloimmune and autoimmune disorder that occurs following allogeneic hematopoietic stem cell transplantation (allo-HSCT), characterized by immune dysregulation, immunodeficiency, impaired organ function, and decreased survival (5), vitamin D deficiency may represent a factor of influence for various cGVHD outcomes. Recent studies have shown that low vitamin D level before allo-HSCT is an independent risk factor for the development of cGVHD (6,7). Also, Silva et al (8) found that the severity of cGVHD assessed according to National Institutes of Health (NIH) response criteria (9) improved in a small group of cGVHD patients who received vitamin D supplementation for osteoporosis or osteopenia. The 2014 NIH cGVHD Consensus Project Ancillary and Supportive Care guidelines prescribe yearly monitoring of serum 25(OH)D within the recommendations for prevention and management of osteoporosis (10).

Still, there is a lack of knowledge on the role of vitamin D in modulating immunological functions in the cGVHD setting, especially in patients with long-standing moderate or severe cGVHD who have failed many lines of therapy. Patients with cGVHD represent a unique population with complex and multiple factors influencing vitamin D levels. The risk of vitamin D inadequacy in cGVHD is increased since patients are routinely instructed to minimize sun exposure to avoid the exacerbation of cutaneous GVHD (10). Furthermore, glucocorticoid therapy, reduced skin synthesis, lower dietary intake, malabsorption, and/or inadequate hepatic and renal hydroxylation to vitamin D active metabolites are additional risks for insufficient levels of vitamin D (1). This study was undertaken to identify the factors associated with vitamin D status, and determine its potential association with cGVHD disease outcomes defined by the NIH criteria.

## Patients and methods

This cross-sectional study included patients enrolled in an ongoing National Institutes of Health protocol, Prospective Assessment of Clinical and Biological Factors Determining Outcomes in Patients with Graft-vs-Host Disease (clinicaltrials.gov identifier: NCT00092235) between October 2004 and May 2015. All patients signed National Cancer Institute Institutional Review Board-approved consent and reported for a 1-week comprehensive multidisciplinary evaluation, which included subspecialist examinations in dentistry, dermatology, gynecology, ophthalmology, pain and palliative care, rehabilitation medicine, and hematology/oncology. Disease assessment was done using the NIH cGVHD diagnostic and staging system (11). Additionally, patients underwent nutritional evaluation by a certified nutritionist, using Patient-Generated Subjective Global Assessment (PG-SGA) malnutrition screening tool recommended by the American Society of Parenteral and Enteral Nutrition in cancer patients (12). For each individual, a total PG-SGA score was calculated and a PG-SGA global rating was established (well-nourished, moderately or suspected of being malnourished, or severely malnourished) (13). Furthermore, self-reported, recent (over the past month) or current supplement use was recorded (none, vitamin D, multivitamin, both). Blood was drawn for chemistry panels, complete blood counts, and other routine laboratory tests, including serum 25(OH)D. Serum 25(OH)D was measured by liquid chromatography-tandem mass spectrometry from October 2004 to January 2014 (Mayo Clinic Laboratories, Rochester, MN, USA, 2004-2010, and Department of Laboratory Medicine, Clinical Center, NIH, USA, January 2011-2014), and chemiluminescence immunoassay (DiaSorin, Stillwater, MN, USA) effective January 29, 2014 (Department of Laboratory Medicine, Clinical Center, NIH, USA). Vitamin D deficiency was defined as serum 25(OH)D concentration ≤20 ng/mL (1).

Potential factors influencing vitamin D level in our patient population included several sets of variables: age, sex, race (self-reported: African-American vs other), body mass index (BMI), blood drawing season classified in an ordered fashion: winter (December through February), spring and fall (March through May and September through November), and summer (June through August), supplement intake, and physical activity. Supplement intake association with 25(OH) level was assessed in several categories: none vs vitamin D and/or multivitamin intake (any); none or only multivitamin intake vs vitamin D or vitamin D and multivitamin intake; and none or multivitamin only or vitamin D only vs both vitamin D and multivitamin intake. Physical activity was assessed using scores of PG-SGA activities and function evaluation (mostly inactive vs fairly normal activity) (13) as well as the Human Activity Profile (maximal activity score HAP MAS, and adjusted activity score HAP AAS) patient-reported questionnaire of energy expenditure or physical fitness (14).

We also considered transplant characteristics including total body irradiation (yes vs no), intensity of conditioning (myeloablative vs non-myeloablative/reduced intensity conditioning), HLA match (match vs mismatch), donor relationship (related vs unrelated), indication for allo-HSCT, stem cell source (bone marrow, peripheral blood, or cord blood), and time since transplant.

Finally, we considered a number of variables that reflected cGVHD activity and severity including time since diagnosis of cGVHD, history of acute GVHD, body surface area of deep and dermal sclerotic skin involvement (defined as percentage of total body surface area), serum markers of inflammation (platelet count, C3 and C4 complement components, C-reactive protein [CRP], and albumin), number of prior systemic immunosuppressive therapies, intensity of current immunosuppression (defined per Mitchell et al (15) as none, mild = single-agent prednisone <0.5 mg/kg/d, moderate = prednisone ≥0.5 mg/kg/d and/or any single agent/modality, and high = 2 or more agents/modalities ± prednisone ≥0.5 mg/kg/d), and current glucocorticoid dose converted to equivalent prednisone dose. Renal function was assessed by estimation of creatinine clearance (eCCr) using the Cockroft-Gault equation (16).

Chronic GVHD outcomes assessed for association with vitamin D included NIH global score (11), average NIH organ score (sum of all NIH organ scores divided by number of organs assessed; 7 for men, 8 for women), NIH Lung Score (11), number of organs affected, cGVHD activity (defined by clinician impression: inactive vs active and highly active), GVHD Physician Global Symptom Severity Score (9), and Lee Symptom Scale Total Score (17). Follow-up for survival analysis was carried out beginning with the date of entry onto the protocol until death or last follow up.

### Statistical analysis

Univariate associations between a set of parameters and continuous or categorized vitamin D level (≤20 vs >20 ng/mL) were initially determined prior to performing multivariable analyses. Statistical methods used in these univariate analyses included the following: exact Wilcoxon rank sum test, Kruskal-Wallis test, Fisher exact test, Mehta’s modification to Fisher exact test (18), Jonckheere-Terpstra trend test (19), Cochran-Armitage trend test (20), and Spearman rank correlation. Spearman correlations were interpreted as follows: r >0.70 = strong correlation; 0.5<r <0.7 = moderately strong correlation; 0.3<r <0.5 = weak to moderately strong correlation; r <0.3 = weak correlation. In view of the large number of exploratory analyses performed to determine factors associated with vitamin D or vitamin D≤20 ng/mL, only *P* < 0.005 was considered statistically significant. Following the initial univariate screening analyses, multiple logistic regression analyses were used to determine the association between parameters and categorized vitamin D. Analyses to determine the association between continuous vitamin D level, as well as categorized vitamin D level (≤20 vs >20 ng/mL; <50 vs ≥50 ng/mL; and ≤20 vs 20-50 vs ≥50 ng/mL as ordered categories) and a variety of cGVHD outcomes were performed. Kaplan-Meier analyses and log-rank tests were used to determine the association between potential predictors and survival after entering on the trial. Beginning with univariate parameters with *P* < 0.050 in the initial screening for association with vitamin D level, multiple logistic regression was used to develop a predictive model for low vitamin D. All *P*-values are two tailed and reported without any adjustment for multiple comparisons. Analyses were performed using SAS version 9.3 (SAS Institute, Inc., Cary, NC, USA).

## Results

### Patients

Initial screening was performed on 364 patients; 17 patients who were not diagnosed with cGVHD at evaluation or who failed to complete the study, 29 pediatric patients, and 8 patients who lacked 25(OH)D measurement were excluded. The final sample consisted of 310 adult participants.

The median age was 48 years (interquartile range [IQR], 36-57) and 172 participants were men (55%). The most frequent indications for transplant were acute leukemia and myelodysplastic syndrome (47%), followed by lymphoma (24%). Myeloablative conditioning was more common (55%) than reduced intensity conditioning (45%). Patients predominantly received peripheral blood stem cells (82%), usually from HLA-matched donors (84%), and 188 patients (61%) were related to their respective donors (Table 1).

**Table 1:** Demographic characteristics of patients with with chronic graft-vs-host disease

	N (% or interquartile range)
All	310
Median age in years	48 (36-57)
Sex	
male	172 (55)
female	138 (45)
Disease	
acute lymphocytic leukemia, acute myeloid leukemia, myelodysplastic syndrome	146 (47)
chronic myeloid leukemia, idiopathic myelofibrosis, myeloproliferative disorder	40 (13)
chronic lymphocytic leukemia	22 (7)
Hodgkin’s lymphoma, non-Hodgkin’s lymphoma	74 (24)
multiple myeloma	15 (5)
aplastic anemia, paroxysmal nocturnal hemoglobinuria	7 (2)
other*	6 (2)
Conditioning regimen	
myeloablative	169 (55)
non-myeloablative	139 (45)
unknown	2 (<1)
Stem cell source	
bone marrow	53 (17)
peripheral blood stem cells	253 (82)
cord	4 (1)
Donor relationship	
related	188 (61)
unrelated	120 (39)
unknown	2 (<1)
Human leukocyte antigen match	
yes	260 (84)
no	43 (14)
unknown	7 (2)

*Other includes Ewing’s sarcoma, essential thrombocythemia, Kostmann syndrome sickle cell anemia, and Wiskott-Aldrich syndrome.

Patients were diagnosed with cGVHD a median of 223 days (IQR, 145-373) after transplant and were enrolled a median of 1105.5 (IQR, 692-1819) days after transplant. Most patients had severe (72%), followed by moderate (26%), cGVHD assessed by NIH global scoring. The median average NIH organ score was 1.13 (IQR, 0.75-1.43) with a median of 5 affected organs (IQR, 4-6). Patients were most frequently on high (39%) or moderate (37%) intensity immunosuppression and had failed a median of 4 prior systemic therapies (IQR, 2-5; Table 2).

**Table 2:** Chronic graft-vs-host disease (cGVHD) characteristics

	N (% or interquartile range)
All	310
Median days from transplant to cGVHD diagnosis	223 (145-373)
Median days from cGVHD diagnosis to consent	737 (338-1465)
Median days from transplant to consent	1105.5 (692-1819)
cGVHD organ involvement*	
skin	243 (78)
joints and fascia	193 (62)
eyes	250 (81)
mouth	212 (68)
lungs	237 (76)
liver	157 (51)
gastrointestinal tract	139 (45)
genital (females only, N = 138)	80 (58)
average National Institutes of Health (NIH) organ score	1.13 (0.75-1.43)
Number of organs affected by cGVHD	median 5 (4-6)
1-2 affected organs	14 (5)
3-4 affected organs	110 (35)
5-6 affected organs	133 (43)
7-8 affected organs	53 (17)
NIH Global Score^†^	
mild	5 (2)
moderate	81 (26)
severe	224 (72)
Prior cGVHD systemic treatment regimens	
<2	31 (10)
2-3	106 (34)
4-5	113 (36)
>5	59 (19)
unknown	1 (<1)
Intensity of current immunosuppression^‡^	
none/mild	74 (24)
moderate	115 (37)
high	121 (39)

*cGVHD organ involvement per NIH organ staging (11).

†Definition for NIH Global score is as follows: mild (1 to 2 organs affected by chronic GVHD with scores of 1), moderate (more than 2 organs with score of 1, any score of 2, or lung score of 1), or severe (any score of 3 or lung score of 2) (11).

‡Definition of intensity of current immunosuppression is as follows: mild (single-agent prednisone 0.5 mg/kg/d), moderate (single-agent prednisone 0.5 mg/kg/d and/or any single agent/modality), high (2 or more agents/modalities +/− prednisone 0.5 mg/kg/d) (15).

### Serum 25(OH)D levels and determination of associated factors

The median value of serum 25(OH)D for all 310 patients was 30 ng/mL (IQR 22-42). 241 patients (77.7%) had serum 25(OH)D>20 ng/mL and 69 (22.3%) had 25(OH)D≤20 ng/mL.

Significantly more patients with 25(OH)D>20 ng/mL used a vitamin D supplement or a multivitamin compared to those who used no supplementation (*P* < 0.001). Also significantly more patients with 25(OH)D≤20 ng/mL used no supplementation or used multivitamin only compared to patients who were taking vitamin D or both (vitamin D and multivitamin) (*P* < 0.001,Table 3).

**Table 3:** Qualitative parameters and their association with categorized 25(OH)D level

	25(OH)D≤20 ng/mL N (%)	25(OH)D>20 ng/mL N (%)	*P*
Race			
African-American	7 (53.8)	6 (46.2)	0.011*
non-African-American	62 (20.9)	234 (79.1)	
Vitamin D supplementation			
none	46 (34.6)	87 (65.4)	<0.001*
vitamin D and/or multivitamin	23 (13.1)	152 (86.9)	
none or multivitamin	58 (28.7)	144 (71.3)	<0.001*
vitamin D or vitamin D and multivitamin	11 (10.4)	95 (89.6)	
none or multivitamin or vitamin D	66 (24.9)	199 (75.1)	0.009*
vitamin D and multivitamin	3 (7.0)	40 (93.0)	
Patient-Generated Subjective Global Assessment			
severely malnourished	10 (55.6)	8 (44.4)	**0.004†**
moderately malnourished	20 (23.8)	64 (76.2)	
well-nourished	38 (19.4)	158 (80.9)	
moderately/severely malnourished	30 (29.4)	72 (70.6)	0.059*
well-nourished	38 (19.4)	158 (80.6)	

*Fisher exact test.

†Cochran-Armitage trend test.

Overall, 175 (56%) patients reported recent or current supplement use, and they had significantly higher 25(OH)D levels than non-users; *P* < 0.001). Patients who reported vitamin D or vitamin D and multivitamin intake had significantly higher 25(OH)D levels than patients who were taking no supplements or multivitamin only (*P* < 0.001). Patients who reported taking both supplements (vitamin D and multivitamin) had significantly higher 25(OH)D levels than patients who were taking no supplements or multivitamin only or vitamin D only (*P* < 0.001; Table 4).

**Table 4:** Qualitative parameters and their association with actual 25(OH)D level

	25(OH)D ng/mL(median, interquartile range)	*P*
Sex		
male	28 (21-36.5)	0.009*
female	31.5 (24-45)	
Race		
African-American	18 (14-35)	0.093*
non-African-American	30 (23-42)	
Vitamin D supplementation		
none	25 (17-32)	<0.001*
vitamin D and/or multivitamin	34 (26-45)	
none or multivitamin	26 (20-36)	<0.001*
vitamin D or vitamin D and multivitamin	34.5 (27-48)	
none or multivitamin or vitamin D	28 (21-39)	<0.001*
vitamin D and multivitamin	39 (30-52)	
Patient-Generated Subjective Global Assessment Physical Activity and Function		
inactive	27.5 (19-39)	0.055*
active	31 (23-43)	
Disease		
acute myeloid leukemia, acute lymphocytic leukemia, myelodysplastic syndrome	31 (23-44)	0.099^†^
chronic myeloid leukemia	25 (23-34)	
chronic lymphocytic leukemia	24 (16-35)	
lymphoma	33 (24-43)	
other^§^	29.5 (22-32)	
acute leukemia, myelodysplastic syndrome, or lymphoma	31 (23-43)	0.009*
chronic myeloid leukemia, chronic lymphocytic leukemia, multiple myeloma, and other^§^	26 (20.5-33.5)	

*Wilcoxon rank sum test.

†Kruskal-Wallis test with Monte Carlo estimate for an exact test.

§Other include sarcoma, immunodeficiency, aplastic anemia, paroxysmal nocturnal hemoglobinuria, Ewing’s sarcoma, essential thrombocythemia, sickle cell anemia, and Wiskott-Aldrich syndrome.

There was a significant positive association between PG-SGA nutritional evaluation, categorized in an increasing ordered fashion as severely malnourished compared to suspected or moderately malnourished compared to well-nourished group, and greater serum 25(OH)D in two categories, ≤20 and >20 ng/mL; *P* = 0.004). When nutritional status was dichotomized to the malnourished group, which included both severely malnourished and suspected or moderately malnourished categories, and well-nourished group, the significance of the association between nutritional status with serum 25(OH)D was lost (*P* = 0.059). Also, no significant association between serum 25(OH)D and PG-SGA score was found. Serum 25(OH)D≤20 ng/mL was somewhat more frequent in African American than in non-African American patients (*P* = 0.011, Table 3), with lower actual 25(OH)D in African Americans compared to non-African Americans but not significantly (*P* = 0.093, Table 4). Serum 25(OH)D was somewhat lower in men than women, but the difference was also not significant (*P* = 0.009). Patients who reported being mostly inactive in PG-SGA activities and function section did not have significantly lower 25(OH)D than patients who reported normal and fairly normal activity (*P* = 0.055). With respect to its association with the primary disease, somewhat higher serum 25(OH)D level was associated with having either acute lymphocytic leukemia (ALL), acute myeloid leukemia (AML), myelodysplastic syndrome, Hodgkin's lymphoma, and non-Hodgkin's lymphoma rather than with having chronic myeloid leukemia, chronic lymphocytic leukemia, multiple myeloma, or other diseases with, but the difference was not significant (*P* = 0.009; Table 4).

The association between BMI, age, and blood drawing season and serum 25(OH)D in low, ≤20 ng/mL group was not significant (*P* = 0.869, 0.195, and 0.844, respectively). No significant correlations were found between serum 25(OH)D and laboratory blood values (serum creatinine, alkaline phosphatase, ALT, AST, GGT, platelet count, CRP, albumin, and magnesium).

Serum 25(OH)D was weakly negatively correlated with eCCr (r = -0.22, *P* < 0.001) and somewhat higher eCCr was found in patients with 25(OH)D≤20 ng/mL (median 104.3, IQR 80.5-135.8) than in patients with >20 ng/mL (median of 93.1, IQR 69.0-123.8) (*P* = 0.012). A weak positive correlation was found between 25(OH)D and time from transplant (r = 0.17, *P* = 0.002).

Prednisone dose was not correlated with serum 25(OH)D (r = 0.047, *P* = 0.414) and it did not significantly differ between 25(OH)D≤20 ng/mL (median 0.13, IQR 0-0.36) and >20 ng/mL patients (median 0.10, IQR 0-0.32) (*P* = 0.920). No correlation was found between 25(OH)D and dermal sclerosis, deep sclerosis, or erythema. There was no significant difference with respect to 25(OH)D≤20 or >20 in TBI conditioning, myeloablative conditioning, HLA match, donor relationship, bone marrow, peripheral blood or cord blood cell source, intensity of immunosupression, prior diagnosis of acute GVHD, acute skin, GI or liver GVHD, cGVHD classification at evaluation, or cGVHD onset (data not shown).

### Multivariable logistic model for the prediction of serum 25(OH)D status

Based on univariate analyses with *P* < 0.05, eCCr, race (African American vs other), supplement intake (vitamin D and/or multivitamin), and PG-SGA global assessment (malnourished, suspected or moderately malnourished, and well nourished) were included in a multiple logistic regression model to predict vitamin D status. By backward elimination, a predictive model was developed (Table 5). This model accurately predicted 77.9% (53/68) of patients with vitamin D≤20 ng/mL and 65.2% (148/227) of patients with vitamin D>20 ng/mL. This is an improvement over the single most predictive univariate factor that was identified as the use of vitamin D and/or multivitamin supplements.

**Table 5:** Predictive model for vitamin D status in chronic graft-vs-host disease*

Variables	*P*	Classification rule	25(OH)D≤20 ng/mL	25(OH)D>20 ng/mL
Creatinine clearance† (value)	0.001	X = (-0.0101 x Creatinine Clearance) + (1.0376 × Race) + (1.1915 × Vitamin D Supplementation) - (0.6552 × Global PG-SGA Assessment)	Correctly predicts 77.9% (53/68)	Correctly predicts 65.2% (148/227)
Race	<0.001			
African-American (1)				
non-African-American (2)				
Vitamin D supplementation	<0.001			
none (0)				
vitamin D and/or multivitamin (1)				
Patient-Generated Subjective Global Assessment	0.005	If X≤1.3431, predicts 25(OH)D≤20 ng/mL		
severely malnourished (2)				
moderately malnourished (1)		If X>1.3431, predicts 25(OH)D>20 ng/mL		
well-nourished (0)				

*Multiple logistic regression modeling was used to generate models predictive for vitamin D level. Created by backward elimination the resulting model was converted into a classification rule that determines the following relationship.

†Creatinine clearance estimated using Cockroft-Gault equation (16).

### Serum 25(OH)D level association with cGVHD outcomes and survival

No significant association was found between continuous vitamin D level or, categorized vitamin D level as ≤20 compared to >20 ng/mL; <50 compared to ≥50 ng/mL; and ≤20 compared to 20-50 or ≥50 ng/mL as ordered categories, with major cGVHD outcomes including NIH global severity score, NIH average score, number of organs affected by cGVHD, NIH Lung Score, cGVHD activity (active vs inactive), GVHD Physician Global Symptom Severity Score, and Lee Symptom Scale Total Score (data not shown).

**Figure 1:**
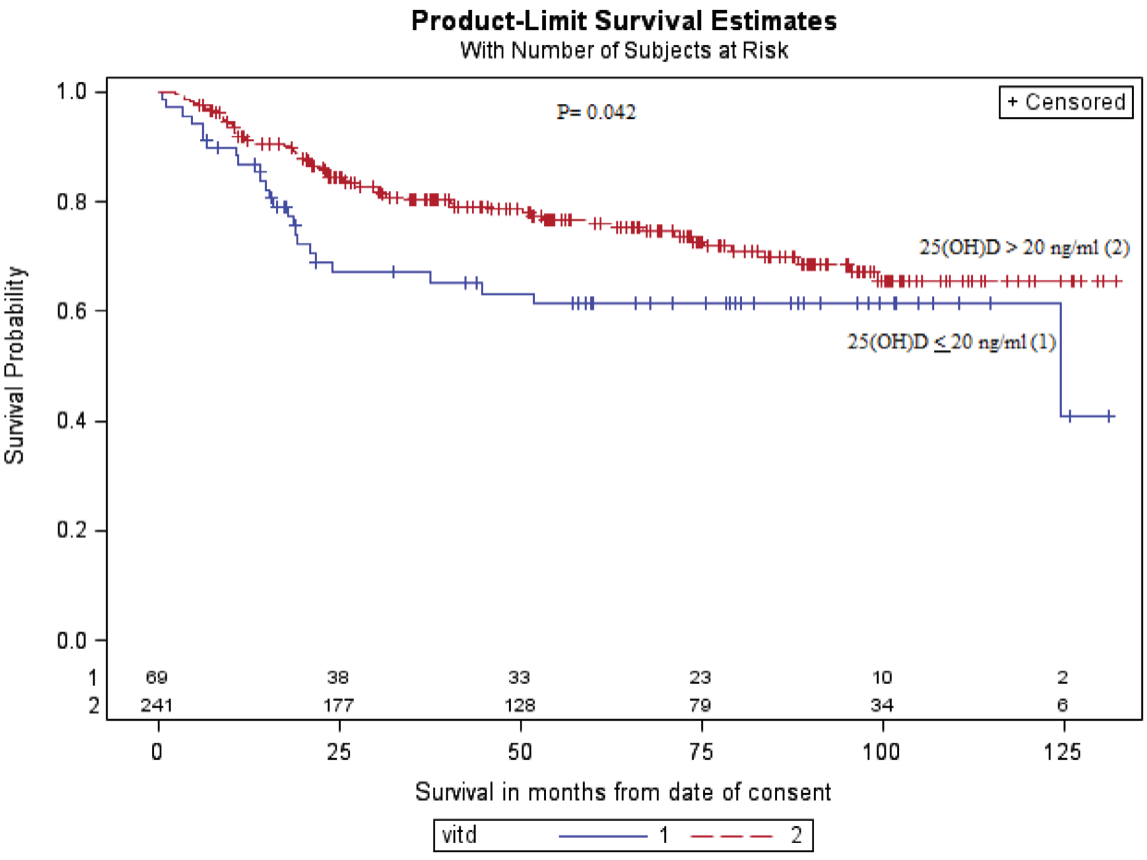
Survival analysis for vitamin D in chronic graft-vs-host disease (cGVHD). Patients with serum 25(OH)D≤20 ng/mL had somewhat poorer survival than patients with serum 25(OH)D>20 ng/mL (*P* = 0.042).

**Figure 2:**
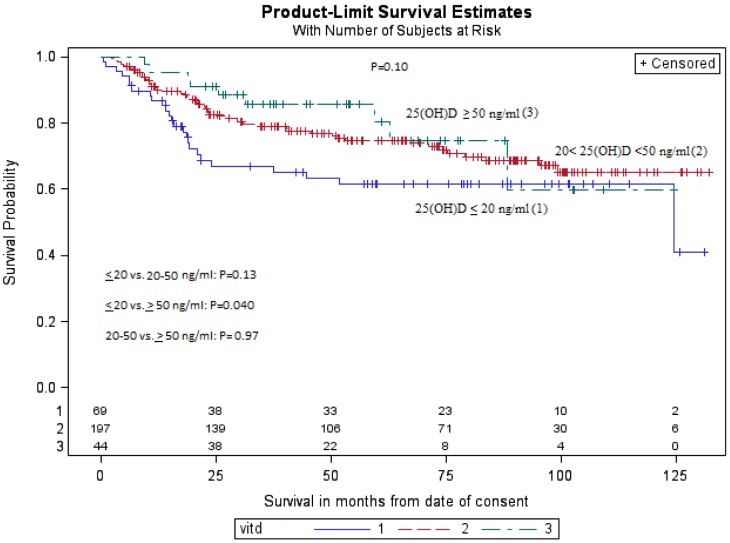
Survival analysis for vitamin D in groups of chronic graft-vs-host disease (cGVHD) patients stratified by serum level of 25(OH)D≤20 vs 20-50 vs ≥50 ng/mL. Patients with 25(OH)D≤20 ng/mL had somewhat poorer survival than patients with 25(OH)D≥50 ng/mL (*P* = 0.040), but no difference in survival was found between groups with serum 25(OH)D in a range of 20-50 ng/mL and those with ≥50 ng/mL

Patients with vitamin D≤20 ng/mL had somewhat decreased overall survival than those with 25(OH)D>20 ng/mL, but the difference was not significant (*P* = 0.042, Figure 1). As a further descriptive evaluation, patients were further divided into the following groups: 25(OH)D≤20 vs 20-50 vs ≥50 ng/mL and their survivals were compared together (Figure 2; global *P* = 0.100). Patients with 25(OH)D≤20 ng/mL had somewhat, but not significantly, worse overall survival than patients with 25(OH)D≥50 ng/mL (*P* = 0.040), but there was no difference in survival between 25(OH)D = 20-50 and 25(OH)D≥50 ng/mL groups (*P* = 0.970).

## Discussion

This study comprising a large group of cGVHD patients showed a significant difference between patients with 25(OH)D level ≤20 and >20 ng/mL in the use of supplements (none, vitamin D and/or multivitamin), and PG-SGA global rating for nutritional status (*P* < 0.005). Also, the univariate screening analyses between patients with 25(OH)D level ≤20 and >20 ng/mL showed an association with race (African-American compared to others) as well as eCCr (*P* < 0.05). The logistic regression model, based on supplement use, race, eCCr and nutritional status, correctly classified 77.9% of participants to have 25(OH)D≤20 ng/mL. This is the first model to our knowledge for predicting the likelihood of vitamin D deficiency in cGVHD patients. The single most predictive factor of vitamin D status was the use of vitamin D and/or multivitamin supplements. This supports the findings of a study where 65% of long-term HSCT survivors had serum 25(OH)D concentrations above 30 ng/mL at least 1 year after HSCT, while 58% of all participants reported regular use of vitamin D supplements (21). Regular or occasional use of supplements could also explain our observation of a weak positive correlation between 25(OH)D with the time from transplant. Association between low vitamin D and more recent allo-HSCT was also reported in patients with AML and ALL, most of whom had GVHD (22).

In our study, African-American race was associated with low vitamin D level, similar to studies in the general population, which showed that African Americans were at high risk for vitamin D insufficiency and all-cause mortality at lower 25(OH)D concentrations than whites (23,24).

In the general population, higher BMI is associated with vitamin D deficiency (25). In our study, higher BMI was not significantly associated with lower vitamin D, contrary to studies in long-term adult survivors of HSCT (21), patients undergoing HSCT (26), or cancer patients (27-29). Furthermore, we did not find an association between 25(OH)D and serum albumin, a routine marker of undernutrition. However, we found an association between serum 25(OH)D levels ≤20 ng/mL and severe and moderate malnutrition assessment per global PG-SGA rating. A recent study on malnutrition in patients with cGVHD observed a mismatch between BMI and nutritional evaluation per global PG-SGA rating, showing that BMI is not a reliable measure of nutritional status in cGVHD setting (30). Taken together, our findings support the use of PG-SGA global assessment for nutritional evaluation of cGVHD patients in detecting severely and moderately malnourished patients who are at a greater risk of vitamin D deficiency.

We observed a previously not reported higher eCCr in patients with 25(OH)D≤20 and negative correlation between eCCr and serum 25(OH)D. It has been reported that corticosteroids and vitamin D metabolites probably modify the production rate and the release of creatinine, although treatment with calcitriol does not appear to change glomerular filtration rate (GFR) (inulin clearance) (31). A study on nondialyzed patients with chronic renal failure found that eCCr positively correlated with serum levels of 1,25(OH)2D. No correlation was observed with 25(OH)D, but the levels of [24,25(OH)2D] were significantly associated with 25(OH)D, presumably largely via the activation of extrarenal 25(OH)D 24-hydroxylase (32), which represents the catabolic pathway of vitamin D where both 1,25(OH)2D and 25(OH)D are metabolized to the water-soluble biologically inactive calcitroic acid (33). It has been shown that glucocorticoids affect vitamin D metabolism by activating the conversion of 25(OH)D and 1,25(OH)2D to calcitroic acid (33), and also that acute and chronic administrations of glucocorticoids increase GFR (34). Contrary to expected, our results did not show an association between serum 25(OH)D with prednisone dose, although the majority of patients were on high (39%) and moderate (37%) intensity immunosuppression. In contrast to our results, Robien et al (21) found prednisone level to be significantly inversely associated with serum 25(OH)D in long-term survivors of HSCT. 80% of their participants unlike in our study, were not on prednisone therapy at the time, and less than half had a history of acute or chronic GVHD. We speculate that severe cGVHD patients on long-term immunosuppression, which could be associated with increased eCCr, may have impaired vitamin D metabolism in terms of increased vitamin D catabolism and conversion to its inactive form.

Although serum 25(OH)D levels may function as a vitamin D biomarker of exposure, it is not clearly established which 25(OH)D levels serve as a biomarker of effect (2). When we used a cut-off value of 20 ng/mL, there were no significant differences with any of the major cGVHD outcomes and these two levels of vitamin D. Currently, some authors claim that the preferred range of serum 25(OH)D is 40-60 ng/mL (35). However, our study found no significant differences in major cGVHD clinical outcomes criteria between groups with serum 25(OH)D<50 and ≥50 ng/mL as well as among groups with serum 25(OH)D≤20 vs 20-50 vs ≥50 ng/mL.

Due to the fact that this was a cross-sectional study with a longitudinal survival follow-up, we were not able to observe the change in cGVHD activity and severity pre and post vitamin D supplementation. Also, limitations of this study include the absence of a control group of patients after allo-HSCT without cGVHD. However, patients with vitamin D≤20 ng/mL had somewhat decreased overall survival (*P* = 0.042). A recent study by Wallace et al (36) in children showed an association between vitamin D<20 ng/mL at 100 days post-HSCT and decreased overall survival, with no difference in survival between children with vitamin D levels above and below 30 ng/mL at day 100. Another recent study HSCT found significant association between serum 25(OH)D<10 ng/mL with overall survival when adjusted for age ≥40 (7). Our observations at different cutoff points suggest that serum 25(OH)D≤20 ng/mL may be associated with decreased survival in cGVHD patients. Finally, this study was prone to referral center bias. The NIH/NCI natural history of cGVHD study primarily enrolled patients who had failed several lines of therapy and sought expert opinion and additional options. However, these are patients with the greatest burden of the disease and associated disability who may most benefit from timely preventive and intervention strategies.

Although increasing evidence supports the role of vitamin D in modulating the immune system, in the context of cGVHD setting many details still remain unknown. Intervention studies are needed to evaluate the best strategies of timely and adequate vitamin D supplementation in the setting of allo-HSCT and cGVHD, as well as to reveal underlying mechanisms of vitamin D metabolism in the complex cGVHD setting.
